# Dystrophie maculaire juvénile de Best

**DOI:** 10.11604/pamj.2014.19.79.4947

**Published:** 2014-09-25

**Authors:** Ryme Abdelkhalek, Abdelbar Oubaaz

**Affiliations:** 1 Service Ophtalmologie, Hôpital Militaire d'Instruction Mohamed V, Rabat, Maroc

**Keywords:** Dystrophie maculaire, acuité visuelle, angiographie, macular dystrophy, visual acuity, angiography

## Image en medicine

Enfant âgé de 10 ans, sans antécédents particuliers, qui présente une baisse d'acuité visuelle progressive évoluant depuis 3mois, chez qui l'examen du fond d’œil montre une maculopathie au niveau des deux yeux donnant un aspect de fibrose (A, B). Une angiographie à la fluorescéine a montré, au niveau de l’œil droit une trainée d'hyper fluorescence entourant une zone d'hypo fluorescence confirmant l'aspect de fibrose observé au fond d’œil (C), au niveau de l’œil gauche une hyper fluorescence limitée à la lésion, dès le temps précoce qui augmente progressivement d'intensité jusqu'au temps tardif, irrégulière au centre, évoquant une néo vascularisation (D). La tomographie par cohérence optique montre au niveau de l’œil droit un œdème maculaire, un décollement de l’épithélium pigmentaire avec dédoublement du complexe membrane de Bruch / épithélium pigmentaire centré par une zone d'hyper réflectivité (E), au niveau de l’œil gauche un œdème maculaire avec un épaississement irrégulier intéressant les couches de l’épithélium pigmentaire, avec une hyper réflectivité confirmant d'emblée la néo vascularisation observée à l'angiographie (F). Une enquête familiale a été ouverte à la recherche d'une dystrophie maculaire héréditaire. L'examen du père et de la sœur a trouvé au niveau des deux yeux, une maculopathie d'aspect vitelliforme, ce qui est en faveur d'une transmission autosomique dominante. Devant cette maculopathie familiale, et le bilan inflammatoire et infectieux jusque-là normal on a évoqué le diagnostic d'une dystrophie maculaire juvénile de Best.

**Figure 1:**
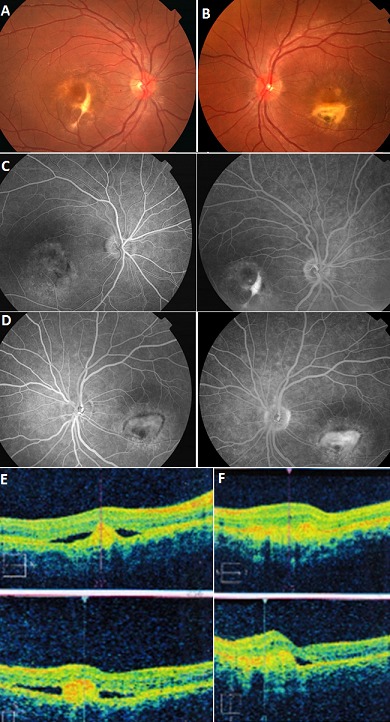
(A): fond d’œil (OD) foyer maculaire donnant un aspect de fibrose; (B): fond d’œil (OG) foyer maculaire blanchâtre ovalaire avec un aspect grisâtre irrégulier au centre; (C): OD: trainée d'hyperfluorescence entourant une zone d'hypofluorescence confirmant l'aspect de fibrose observé au FO; (D): OG: hyperfluorescence limitée à la lésion, dès le temps précoce qui augmente progressivement d'intensité jusqu'au temps tardif, irrégulière au centre, évoquant une néovascularisation; (E): OD: œdème maculaire (épaisseur fovéolaire à 319 µm), un décollement de l'EP avec dédoublement du complexe MB/EP centré par une zone d'hyperreflectivité; (F): OG: œdème maculaire (324µm) avec un épaississement irrégulier intéressant les couches de l'EP, avec une hyperreflectivité confirmant d'emblée la néovascularisation observée à l'AGF

